# Femtosecond laser irradiation has an anti-virulence effect by reducing the adhesion and invasion of MRSA bacteria to human cells: an in vitro study

**DOI:** 10.1007/s10103-025-04664-9

**Published:** 2025-10-25

**Authors:** Esraa Ahmed, Ahmed O. El-Gendy, Michael R. Hamblin, Tarek Mohamed

**Affiliations:** 1 Laser Institute for Research and Applications LIRA, Beni-Suef University, Beni-Suef, Egypt; 2 University of Johannesburg, Doornfontein, South Africa

**Keywords:** Antibacterial, Photodynamic therapy, Biofilm, Laser, Bacterial adhesion, Bacterial invasion

## Abstract

**Purpose:**

Multidrug-resistant bacterial infections are a substantial global health challenge. Targeting bacterial virulence factors that control the progression, severity, and pathogenicity of bacterial infections could prevent and combat infection by intractable, potentially life-threatening pathogens such as methicillin-resistant *Staphylococcus aureus* (MRSA). Laser-based antibacterial photodynamic therapy (aPDT) has emerged as a promising alternative for infection management.

**Methods:**

This study assessed the attenuation of adhesion and invasion of MRSA bacteria in two human epithelial cell lines, melanoma cells (A375) and breast ductal carcinoma (T47D), after exposure to different sublethal femtosecond laser doses. The INSPIRE HF100 laser system (Spectra Physics), pumped by a mode-locked femtosecond Ti: sapphire laser MAI TAI HP (Spectra Physics), was used to provide the femtosecond laser pulses at a wavelength of 400 nm for different durations; 15, 30, and 45 min., as well as different average powers; 50, 100, and 150 mW.

**Results:**

Our results showed that, an average power of 50 mW for 15 min. significantly reduced MRSA adhesion (by ~ 84–96%) and invasion (by ~ 84–98%), even though exposure durations of 30 and 45-min. resulted in reduced viability (*p* < 0.0001 by ANOVA and Tukey test).

**Conclusion:**

All proposed femtosecond laser doses effectively impaired MRSA’s ability to adhere to and invade epithelial cells.

**Graphical Abstract:**

This diagram illustrates the experimental workflow for identifying the optimal **femtosecond laser** parameters to attenuate **MRSA’s adhesion to and invasion of** different epithelial cell lines. Following exposure to femtosecond laser irradiation, MRSA was co-cultured with epithelial cells for various infection durations. Adhesion and Invasion assays were done, and the adherent and invasive MRSA were quantified by colony-forming unit (CFU) counting, allowing for recommending an optimal femtosecond laser treatment for mitigating bacterial adhesion and invasion.

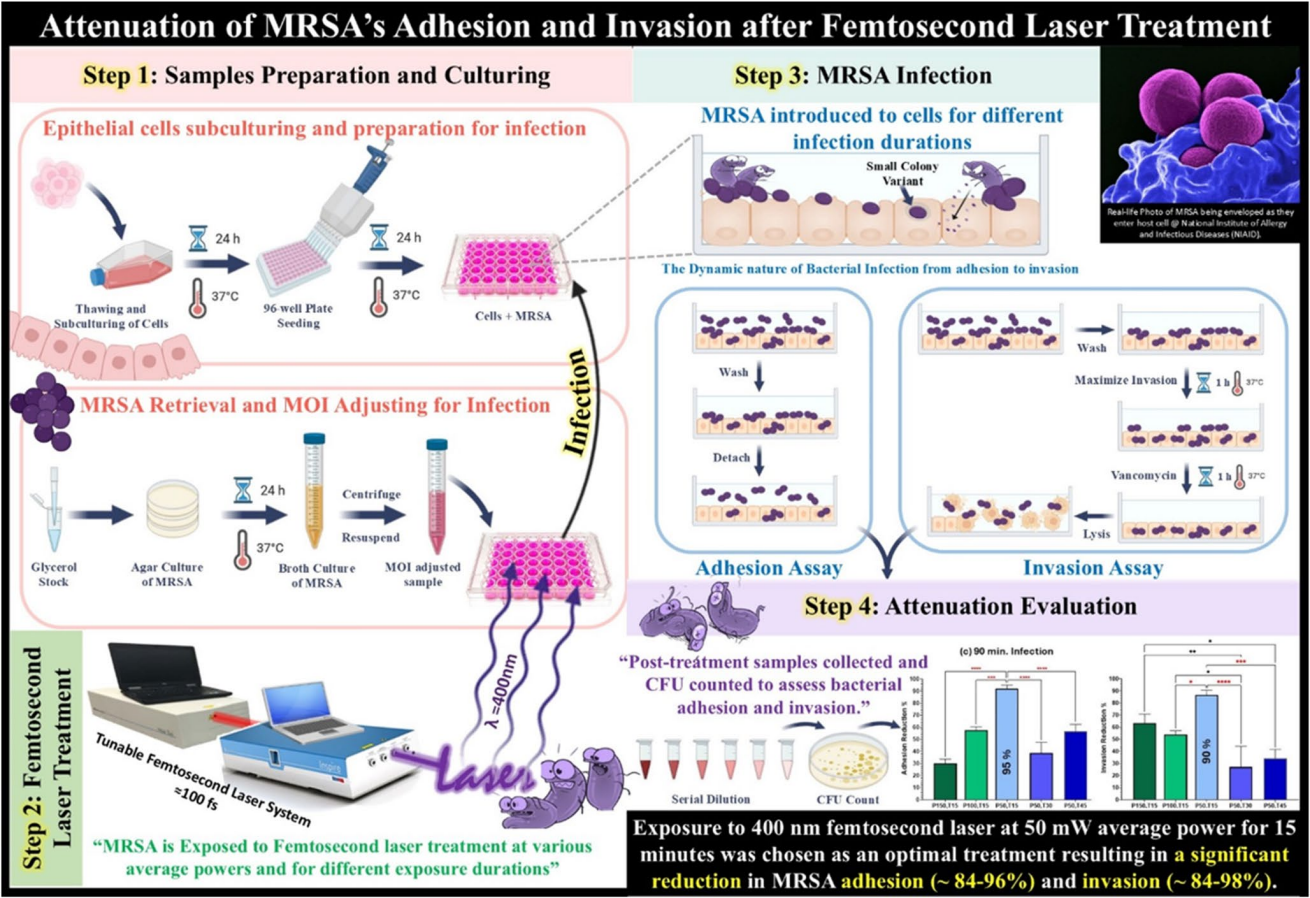

## Introduction

The increasing emergence of multidrug-resistant bacteria presents an alarming global challenge to healthcare as well as an economic burden [1–3]. This is partly caused by the rampant overuse of antibiotics without considering their clinical need [4]. Of these bacteria, methicillin-resistant *Staphylococcus aureus* (MRSA) stands out as a critical opportunistic pathogen, which has colonized more than 30% of the human population [5] causing both superficial and invasive, potentially life-threatening infections [6].

The progression, severity, and pathogenicity of bacterial infections are mainly controlled by specific substances produced by bacteria known as “virulence factors”. These can be cell-associated or secreted following infection, and often exhibit strain-specific characteristics with significant diversity [7]. In contrast to conventional antibiotics, which target bacterial viability, another promising antibacterial strategy aims to eradicate bacteria by suppressing their virulence factors, subsequently facilitating their elimination by the host immune system [8]. *Staphylococcus aureus* (*S. aureus*) can produce several virulence factors, including cell wall-anchored (CWA) proteins. These proteins are attached to the cell wall peptidoglycan on the bacterial surface, and play many crucial roles in bacterial pathogenicity, such as adhesion to and invasion of host cells and tissues [6]. Understanding and targeting these virulence factors, as well as their functions, could be promising to prevent and combat *S. aureus* infections [9, 10].

The intricacy of bacterial adhesion to host cells was first recognized in 1908 [11]. The adhesion of both commensal and pathogenic bacteria is typically facilitated by surface molecules known as adhesins. Gram-positive bacteria, including *S. aureus*, possess many cell wall-associated adhesins known as “*m*icrobial *s*urface *c*omponents *r*ecognizing *a*dhesive *m*atrix *m*olecules**”** (MSCRAMMs). By binding to specific receptors, these adhesins facilitate bacterial interaction with host cells, the extracellular matrix, or other components [12, 13]. Bacterial adhesion to host cells is often a preliminary step for establishing acute or chronic infections. After adhesion, bacteria begin to colonize, grow, and develop into multicellular microbial communities, referred to as biofilms [14]. A biofilm is a heterogeneous 3D growth state where microbial communities are encapsulated in a self-produced protective extracellular polysaccharide matrix along with necrotic tissue [15]. These tangled formations allow bacterial pathogens to bypass the immune system, significantly contributing to infection persistence and higher pathogenicity [16]. Bacterial adhesion was formerly believed to enable bacteria to colonize a site, but then it was realized that adhesion is crucial for bacterial uptake (invasion) and toxin secretion. It can also influence pro-inflammatory or anti-inflammatory responses by modulating innate immune receptors, making it a key process to be studied in bacterial pathogenicity [11].

Various invasive bacterial pathogens have evolved distinct sets of virulence factors, termed invasins, that collectively orchestrate the penetration of cells and/or disrupt the integrity of the junctions between cells, enabling pathogens to infiltrate host tissue [17–19]. *S. aureus* invades the host cells by either making use of cell components, specifically the actin cytoskeleton and its regulatory machinery, or using an indirect approach by interacting with cell surface receptors, such as integrins, which are linked to the cytoskeleton to trigger actin-dependent internalization [20]. The severity of bacterial infections significantly increases when bacterial pathogens infiltrate the host cells. For example, the invasion of the vascular endothelium by *S. aureus* can lead to severe complications such as sepsis, endocarditis, arthritis, and meningitis, all of which are associated with high mortality rates [21–24].

Targeting bacterial adhesion and invasion is the aim of various potential anti-virulence strategies. However, these proposed strategies have drawbacks that hinder their clinical application [25]. One major drawback is that these agents may have a narrow antibacterial spectrum and limited efficacy, rendering them ineffective against many pathogens [26]. Such a drawback underscores the need for an alternative broad-spectrum anti-virulence strategy to effectively target Gram-positive and Gram-negative bacteria [27]. One promising therapeutic modality is laser-based antimicrobial photodynamic therapy (aPDT), a rapid and minimally invasive approach with great potential for eliminating targeted pathogens [28, 29]. aPDT also has a low risk for the development of bacterial resistance due to its multitargeted mode of action, even after multiple cycles of treatment as practically confirmed in a recent study [30].

aPDT often involves the external addition of photosensitizers designed to bind to the bacteria, but another approach relies on the presence of photosensitizers that have been naturally produced by the bacteria. The antibacterial efficacy of aPDT is due to the localized generation of cytotoxic reactive oxygen species (ROS) based on the interaction of laser radiation with matter, converting light energy into chemical energy through two concurrent photochemical processes [31, 32]. One involves electron transfer (type I), while the other involves energy transfer reactions (type II) [33, 34]. This induced oxidative stress ultimately leads to cell death [35–37]. When bacterial pathogens are exposed to sublethal doses of aPDT, the resulting oxidative stress level within the bacterial cells may not be sufficient to kill the bacteria; however, it can alter microbial virulence factors and their expression [28, 29, 38].

The present report describes a way to mitigate MRSA infections by exploiting the potential anti-virulence efficacy of femtosecond laser irradiation. The objectives were: (1) Evaluate the possible attenuation of MRSA adhesion to and invasion of different human epithelial cell lines after exposure to various femtosecond laser doses; (2) Comparatively evaluate and optimize the femtosecond laser-based aPDT for maximum attenuation of virulence. To the best of our knowledge, this is the first study to assess the effectiveness of sublethal doses of femtosecond laser-based aPDT as an anti-virulence strategy to target MRSA adhesion and invasion, aiming to optimize treatment parameters in anticipation of future clinical implementation.

## Materials and methods

### Microorganisms and culture conditions

Methicillin-resistant *Staphylococcus aureus* ATCC 43,300 was cultured in Brain Heart Infusion (BHI) broth and incubated with shaking for 12 h at 37^o^ C. The overnight culture was then centrifuged for 10 min. at 1006 g, washed in PBS, centrifuged again, and resuspended in PBS. An aliquot of 100 µL was resuspended in 1 mL of Dulbecco’s modified Eagle Medium (DMEM) to achieve the desired multiplicity of infection (MOI) of 200:1. Using sterile pipet tips, a volume of 100 µL of the diluted suspension was transferred into wells of a 96-well microtiter plate before laser exposure. All the experimental procedures were performed inside a sterile laminar flow hood (class II biological safety cabinet, MSC-Advantage TM).

### Femtosecond Laser System and Exposure Setup

The femtosecond laser pulses were generated by the INSPIRE HF100 laser system (Spectra-Physics), which was pumped by a mode-locked femtosecond Ti: sapphire MAI TAI HP laser (Spectra-Physics), with ∼ 1.5–2.9 W average power, 80 MHz repetition rate, and a wavelength ranging from 690 to 1040 nm. The spatial profile of the laser beam had a Gaussian distribution with a TEM00 spatial mode and M^2^ < 1.1. The INSPIRE HF100 laser system was operated at the Second Harmonic Generator (SHG) mode to convert the wavelength of 800 nm from the MAI TAI HP into 400 nm.

**Fig. 1:**
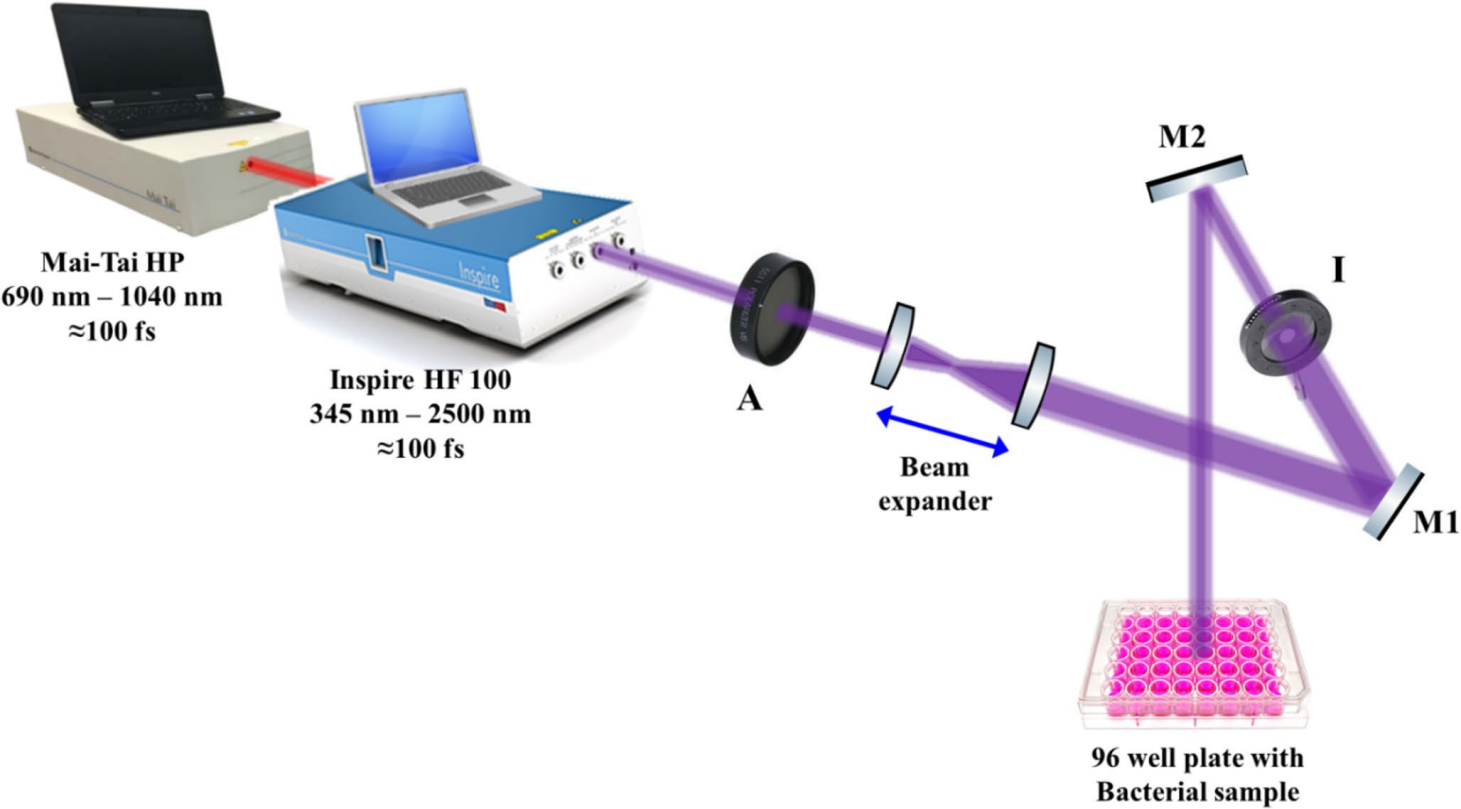
Schematic illustration of the experimental setup for the antimicrobial femtosecond laser treatment. A; Attenuator, M1, and M2; Highly reflective mirrors, I; Iris

To ensure the uniform interaction between laser light and bacterial culture, the laser beam was directed from the output aperture and manipulated to reach the point of application approximately 10 cm above each bacterial culture with the help of various optical and optomechanical components assembled in an optical set up as shown in Fig. 1. Attenuator A was used to adjust the laser power, reaching the samples at selected values of 50, 100, and 150 mW, with a peak power density of 0.63 GW/cm², which were measured using a Newport 843R power meter. A beam expander of two converging lenses was used to expand the initial laser beam diameter from 2 mm to 10 mm. Two highly reflective mirrors, M1 and M2, were used to direct the laser beam onto the samples. The adjustable iris I was used to restrict the diameter of the laser beam to 6 mm, the diameter of each well of the microtiter plate.

### Determination of conditions for sublethal femtosecond laser-based aPDT

This study required using sublethal doses of femtosecond laser-based aPDT to test the possible attenuation of MRSA adhesion and invasion. This sublethal value was previously defined to result in a 1 to 3 log_10_ reduction in colony forming units (CFU)/mL of the pathogen [39–44]. After exposure to femtosecond laser treatments at 400 nm with different average powers 50, 100, and 150 mW, for different exposure durations 15, 30, and 45 min., the samples were then serially diluted (tenfold), plated on BHI agar, and incubated at 37 °C for 24 h. The number of viable MRSA bacteria was defined as CFU/mL.

### Human cell lines and culture conditions

The two human epithelial cell lines, melanoma cells (A375) and breast ductal carcinoma cells (T47D) were used to test the ability of femtosecond laser irradiation to attenuate MRSA adhesion and invasion. Both cell lines were cultured in high glucose, glutamine-containing DMEM (SERANA, Germany) supplemented with 10 % heat-inactivated fetal bovine serum (FBS, Gibco, Thermo Fisher Scientific, USA) and 1% penicillin-streptomycin antibiotic mixture (Gibco, Thermo Fisher Scientific, USA). The cells were cultured and passaged regularly in cell culture flasks (T75 Falcon; Corning) and incubated at 37 °C with 5% CO_2_ and 95% air until reaching confluence, then detached with 0.05 % trypsin (Gibco, Thermo Fisher Scientific, USA), and resuspended in DMEM. For adhesion and invasion assays, 4 × 10^5^ cells/mL were seeded per well in a 96-well plate (Corning, Inc., Corning, NY, USA) and incubated overnight under the same conditions. Before infection, cells were washed twice with pre-warmed sterile PBS and then kept in antibiotic-free cell culture media.

### Bacterial adhesion and invasion assay

The effect of femtosecond laser irradiation in attenuating MRSA adhesion and invasion with different epithelial cell lines was tested as previously described [45–47], with some modifications.

To evaluate MRSA adhesion ability, freshly grown bacterial samples were added (at a MOI of 200:1) to the overnight cultured epithelial cells for different durations of 30, 60, and 90 min. After each incubation, the media was removed, the cell monolayers were washed thrice with pre-warmed PBS to remove non-adherent bacteria, then 10 µL of trypsin/EDTA was added followed by incubation at 37 °C for 90 s to selectively detach the adherent bacteria from the surface of the cells. To stop the effect of trypsin/EDTA 100 µL of prepared medium was added. Tenfold serial dilutions of the detached bacteria were plated on BHI agar and incubated at 37 °C for 24 h. The number of viable adherent MRSA was defined as CFU/mL.

A similar procedure was applied to investigate MRSA invasion ability. Instead of detaching the bacteria after the initial incubation period, cells were washed thrice with pre-warmed PBS and then incubated for an extra period of 60 min. to maximize the invasion of the adherent bacterial cells into the epithelial cells, then they were washed. A fraction of 100 µL of DMEM containing vancomycin (200 µg/mL) was added to each well and incubated for an hour to kill any remaining extracellular bacteria. The cells were then washed, and after that, a fraction of 100 µL of 0.1% Triton X-100 was added into each well and incubated for 5 min at 37 °C to lyse the cells. After plating tenfold serial dilutions on BHI agar and incubation at 37 °C for 24 h, the number of released intracellular MRSA was defined as CFU/mL. Assays were performed in technical triplicates (three wells per condition) across two biological replicates (independent experiments), totaling six measurements per group.

**Fig. 2:**
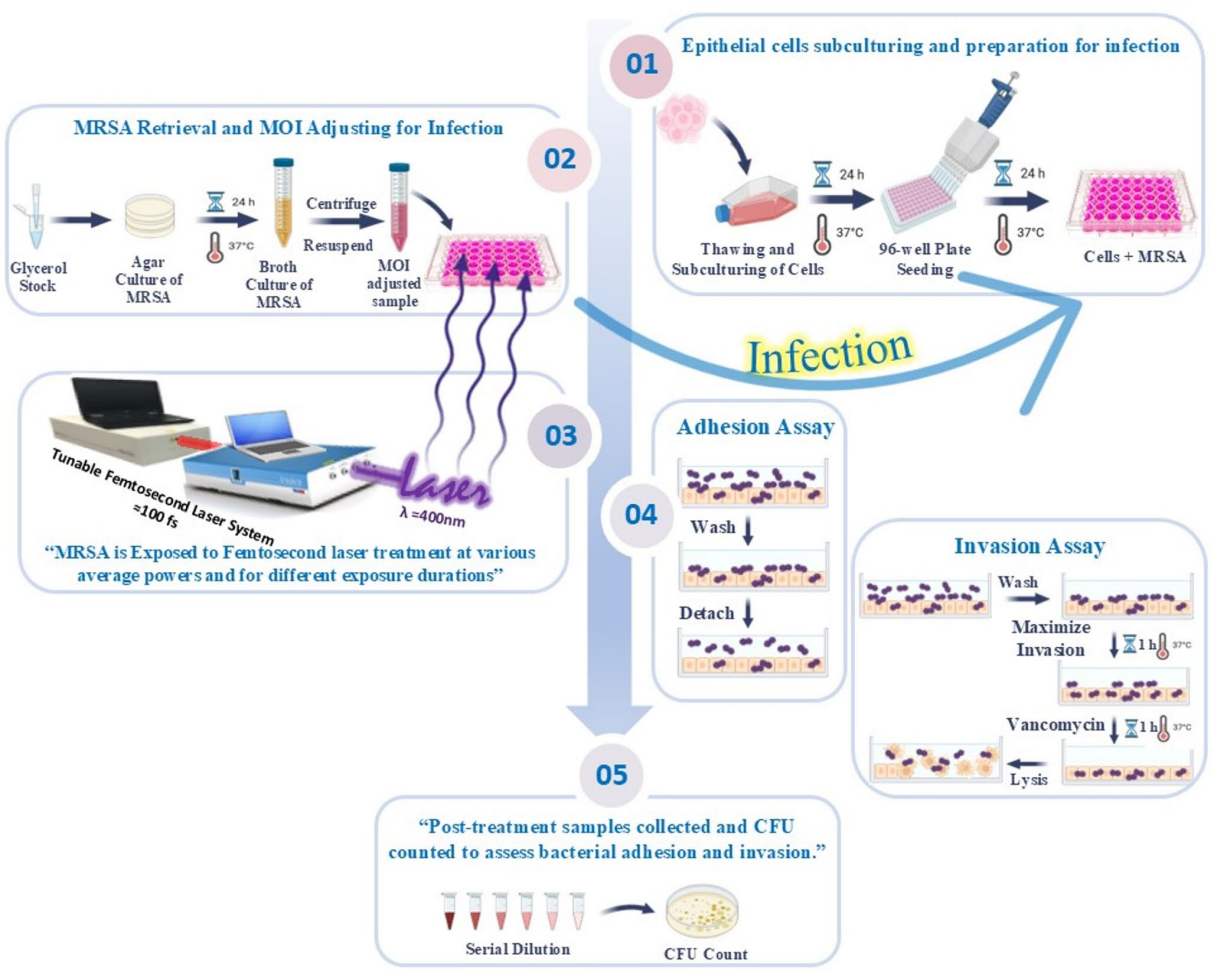
Schematic illustration of the experimental workflow: Testing the attenuation of MRSA adhesion and invasion after different femtosecond laser doses

The experimental procedures are schematically illustrated in Fig. 2.

### Statistical Analysis

Each experiment was performed in triplicate. Bacterial viability counts and percentage reduction were calculated as mean ± standard error. One-way analysis of variance (ANOVA) test followed by Tukey’s test was used to compare the means across different groups using GraphPad Prism 9 software. *p* < 0.05 was considered significant.

## Results and discussion

### Determining the effectiveness of femtosecond laser treatment as an anti-virulence modality

**Fig. 3:**
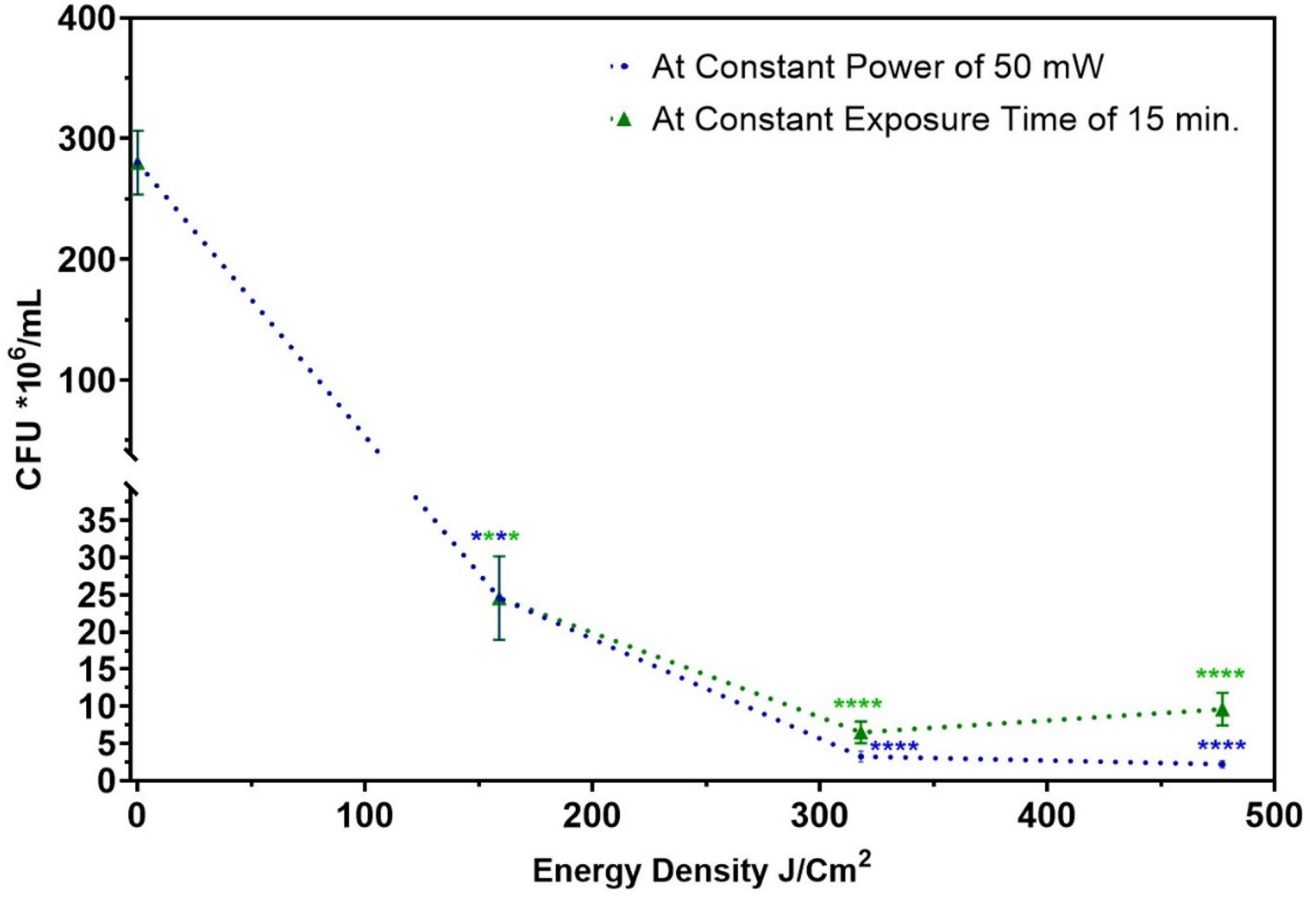
Viability of MRSA as CFU*10^6^/mL after exposure to femtosecond laser irradiation of different energy densities, all at 400 nm. Dotted blue line represents exposure durations at constant average power of 50 mW from 15 min. (energy density of 159 J/cm^2^), 30 min. (energy density of 318 J/cm^2^) and 45 min. (energy density of 477 J/cm^2^). Dotted green line represents varying average power at constant duration of 15 min. from 50 mW (energy density of 159 J/cm^2^), 100 mW (energy density of 318 J/cm^2^) and 150 mW (energy density of 477 J/cm^2^), Dotted lines denote trends only to illustrate dose-dependence; intermediate values were not measured. Statistical significance was determined using ANOVA followed by Tukey’s test (**** *p* < 0.0001)

Targeting MRSA virulence factors can be investigated by exposing the pathogen to sublethal doses of femtosecond laser. Sublethal doses leave sufficient surviving bacteria to test the possible attenuation of MRSA adhesion and invasion in response to different femtosecond laser treatments. In a recent study [48], we examined the antibacterial efficiency of femtosecond laser-based aPDT on the growth kinetics of *S. aureus*, comparing different femtosecond laser parameters to improve the effectiveness of the treatment. We found an average power of 50 mW for 15 min. of exposure to either 390–400 nm (energy density of 159 J/cm^2^) was sufficient to decrease bacterial viability substantially. In the current study, we aimed to increase the proposed energy density by doubling (318 J/cm^2^) and tripling (477 J/cm^2^) the total dose by either increasing the exposure time (from 15 min. to 30 and 45 min.) or the average power reaching the samples (from 50 mW to 100 and 150 mW), at the wavelength of 400 nm, a visible wavelength which is considerably safer for clinical applications than other shorter wavelengths [49, 50]. At this wavelength, the antibacterial effect of laser-based aPDT is mainly due to the photoactivation of the naturally occurring endogenous photosensitizers within the bacterial cells, thought to be metal-free porphyrins [51]. As previously determined in many practical investigations [39–44], a sublethal dose should result in a 1 to 3 log_10_ reduction in the CFU/mL of the pathogen. To ensure that the different femtosecond laser aPDT doses were sublethal, a plot was constructed of viable counts of MRSA (Y-axis) exposed to femtosecond laser irradiation against different energy densities (X-axis) as shown in Fig. 3.

We found that exposure to an average power of 50 mW for 45 min. was near the edge of the sublethal dose limit. A notable observation was that, for each energy density value, increasing the average power at constant exposure time (green line) did not further reduce MRSA viability, while increasing the exposure time while maintaining constant average power (blue line) did further reduce MRSA viability. This could be attributed to two reasons: (1) the phenomenon known as photobleaching, which involves the destruction of the endogenous photosensitizer at high power levels [52]. (2) time-dependent accumulation of ROS where longer exposure times allow for more ROS generation, even at constant power, which highlights the importance of optimizing laser-based aPDT treatment parameters. A similar behavior was reported in our previous study [48], Photobleaching occurred when high fluence was achieved via power escalation (e.g., 150 mW for 15 min), where elevated peak intensities degraded porphyrins. In contrast, fluence increases via longer exposure (e.g., 50 mW for 45 min) favored ROS accumulation.

### Evaluating the attenuation of MRSA adhesion to two human epithelial cell lines after exposure to different femtosecond laser doses

**Fig. 4:**
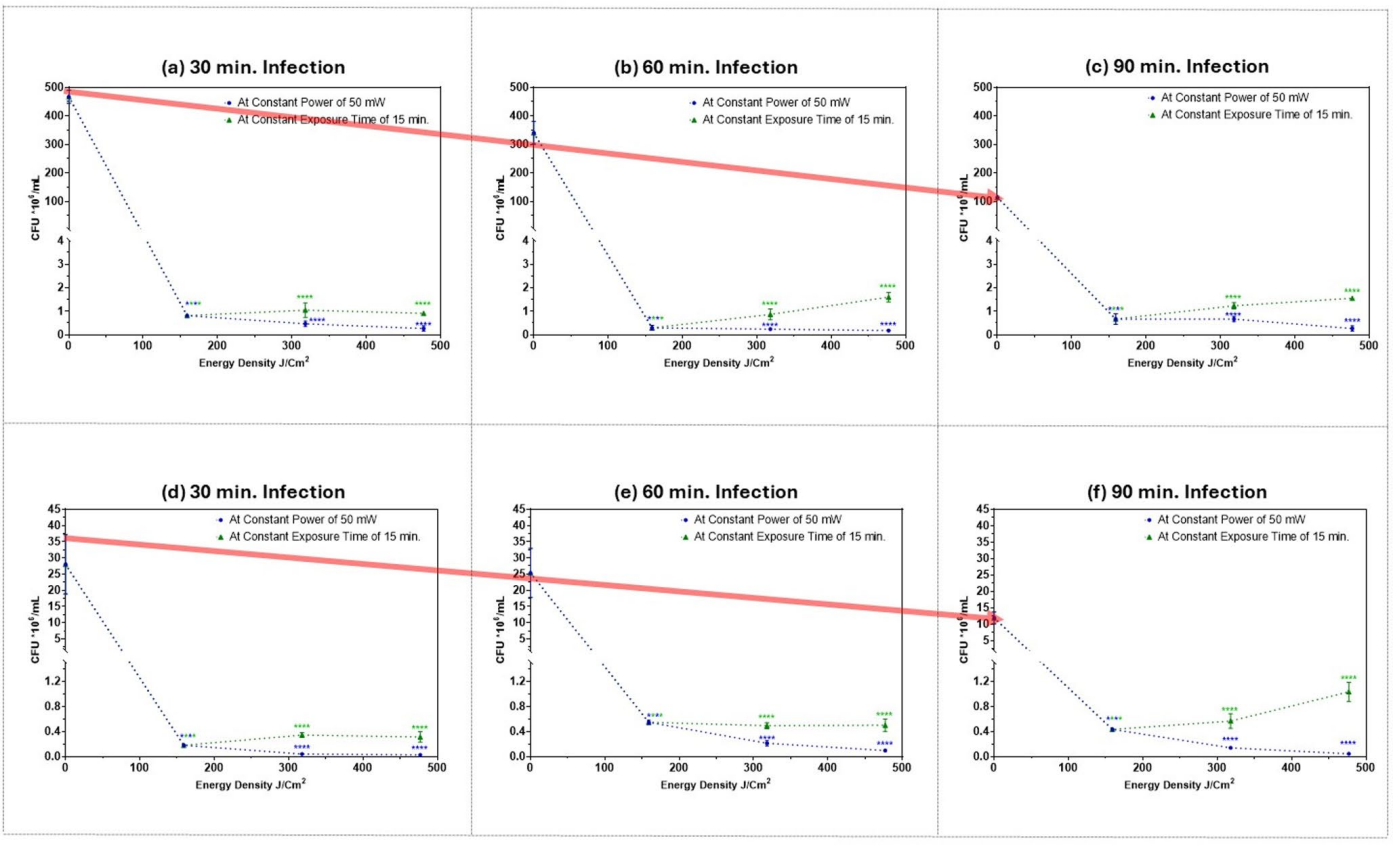
Number of adherent MRSA bacteria (CFU*10^6^/mL) on the surface of human epithelial cell lines: (**a**), (**b**) & (**c**), A375 melanoma cells; (**d**), (**e**) & (**f**) T47D breast ductal carcinoma cells after exposure to 400 nm femtosecond laser irradiation of different energy densities. Dotted blue lines represent different exposure durations at a constant average power of 50 mW; 15 min. providing energy density of 159 J/cm^2^); 30 min. providing energy density of 318 J/cm^2^; 45 min. providing energy density of 477 J/cm^2^. Dotted green line represents different average powers at constant exposure duration of 15 min.: 50 mW providing energy density of 159 J/cm^2^; 100 mW providing energy density of 318 J/cm^2^; 150 mW providing energy density of 477 J/cm^2^. (**a**) & (**d**) 30 min. infection; (**b**) & (**e**) 60 min. infection; (**c**) & (**f**) 90 min. infection, Dotted lines denote trends only to illustrate dose-dependence; intermediate values were not measured. Statistical significance was determined using ANOVA followed by Tukey’s test (**** *p* < 0.0001). Red arrows: highlight baseline control (0 J/cm²) across panels to highlight the dynamic nature of bacterial infection over time. These annotations guide visual comparison without implying statistical significance

In Fig. 4, the number of adherent MRSA bacteria (Y-axis) after exposure to the selected femtosecond laser doses of different energy densities compared to the control sample (X-axis) is plotted. The green line represents an increase in average power at a constant exposure duration and the blue line represents an increase in exposure duration while maintaining constant average power, in both cases providing the same energy density. MRSA adhesion was tested with two human epithelial cell lines; A375 melanoma cells and T47D breast ductal carcinoma cells. As can be seen all treatment regimens reduced the number of viable adherent MRSA bacteria at all the different infection durations: 30 min. (a) & (d), 60 min. (b) & (e), and 90 min. (c) & (f).

When comparing the two cell lines shown in Fig. 4, the number of MRSA adherent to T47D cells was lower when compared to adherent MRSA to A375 cells. This difference could be attributed to the complex physicochemical forces of the bacterial adhesion process, involving both nonspecific hydrophobic and electrostatic forces, as well as specific receptor-ligand interactions that very likely differ between different human epithelial cells [13]. It should be noted that human epithelial breast cells are not a common site of bacterial infection in general. Hence, the reason that MRSA has a higher affinity for binding to A375 cells than to T47D cells could be either due to a stronger or more efficient interaction between MRSA adhesins and the surface receptors on A375 cells compared to T47D cells, or else that A375 cells have more receptors for MRSA adhesins, resulting in better adhesion.

The significance of bacterial adhesion as the initial stage in any bacterial infection highlights the importance of studying its attenuation as a therapeutic strategy. Adhesion provides numerous advantages to bacteria: (1) it enables acute colonization by facilitating access to essential nutrients; (2) when the adhesins interact with the host cell they can induce a rearrangement in the actin network, a step needed for bacterial invasion of mucosal barriers or host cells; (3) adhesion is a crucial initial step for contact-dependent delivery of toxins or effectors as adhesion enables bacteria to approach the host cell membrane closely enough for specialized injection systems to penetrate and deliver toxins into the host cytoplasm. These toxins can disturb the metabolism of the host cells, reprogram the cell to uptake the bacteria, and impair or eliminate the host immune defenses [12].

Adhesins do not generally have a direct harmful effect on the host cells, but one potential consequence of adhesion is inflammation. Certain adhesins directly trigger inflammatory pathways, promoting immune cell infiltration, activation, and phagocytosis, which help clear bacteria; this provoked immune response can affect host cells [11].

### Examining the attenuation of MRSA invasion into two different human epithelial cell lines after exposure to different femtosecond laser doses

**Fig. 5:**
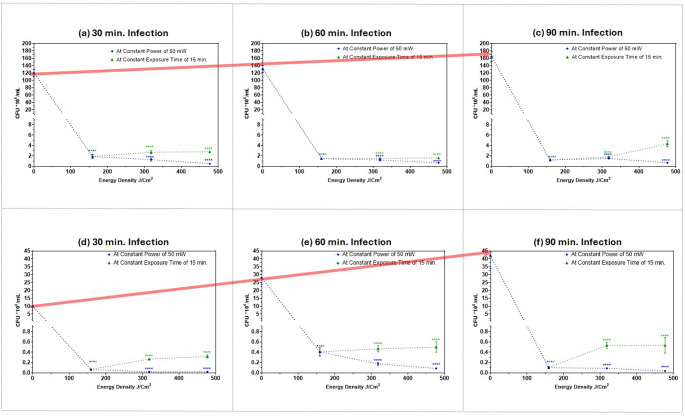
Number of MRSA bacteria (CFU*10^6^/mL) that had invaded into human epithelial cell lines: (**a**), (**b**) & (**c**), A375 melanoma cells; (**d**), (**e**) & (**f**) T47D breast ductal carcinoma cells after exposure to 400 nm femtosecond laser irradiation of different energy densities. Blue lines represent different exposure durations at a constant average power of 50 mW; 15 min. providing energy density of 159 J/cm^2^); 30 min. providing energy density of 318 J/cm^2^; 45 min. providing energy density of 477 J/cm^2^. Green line represents different average powers at constant exposure duration of 15 min.: 50 mW providing energy density of 159 J/cm^2^; 100 mW providing energy density of 318 J/cm^2^; 150 mW providing energy density of 477 J/cm^2^. (**a**) & (**d**) 30 min. infection; (**b**) & (**e**) 60 min. infection; (**c**) & (**f**) 90 min. infection, Dotted lines denote trends only to illustrate dose-dependence; intermediate values were not measured. Statistical significance was determined using ANOVA followed by Tukey’s test (**** *p* < 0.0001). Red arrows: highlight baseline control (0 J/cm²) across panels to highlight the dynamic nature of bacterial infection over time. These annotations guide visual comparison without implying statistical significance

Bacterial invasion is a complex process where pathogens exploit normal host uptake pathways or manipulate cell processes to enter the host cell cytoplasm. Invasion is a property of many specialized pathogenic bacteria. Until recently, *S. aureus* was not considered to be an invasive pathogen, but it was discovered that *S. aureus* can induce its own uptake into non-phagocytic cells [53]. The ability of femtosecond laser to attenuate MRSA invasion into A375 melanoma cells, and T47D epithelial breast ductal carcinoma cells is shown in Fig. 5. The (Y-axis) shows the number of MRSA bacteria that had invaded the cells after exposure to femtosecond laser irradiation with different energy densities (X-axis). The green line represents an increase in average power at a constant exposure duration, while the blue line represents an increase in exposure duration while maintaining constant average power. Both arrangements provided the same energy density. MRSA invasion was investigated at different infection durations: (a) & (d) 30 min.; (b) & (e) 60 min.; (c) & (f) 90 min. All the assessed treatment parameters significantly reduced the number of MRSA bacteria that had invaded the cells.

Bacteria infiltrate host cells to gain several strategic advantages: (1) invasive bacteria are more likely to evade the immune system by concealing their antigens within cells, rendering them unrecognizable by antibodies; (2) the nutrient-rich environment of host cells contains higher concentrations of essential metals, amino acids, and sugars compared to surrounding tissues and fluids; (3) bacteria can exploit mobile phagocytic cells like macrophages, dendritic cells, and neutrophils to spread undetectably throughout the body. This “Trojan horse” mechanism allows bacteria like *Salmonella* to hijack intestinal macrophages, facilitating their spread into the bloodstream [12]. “Getting in” to cells is only half the challenge for bacteria; surviving inside and eventually exiting the host cells is the other half. Once inside cells, bacteria manipulate host signaling processes to repurpose host biology to their advantage, such as by altering the actin cytoskeleton, redirecting metabolism and cell signaling, and inhibiting phagosome pathways [12].

**Fig. 6:**
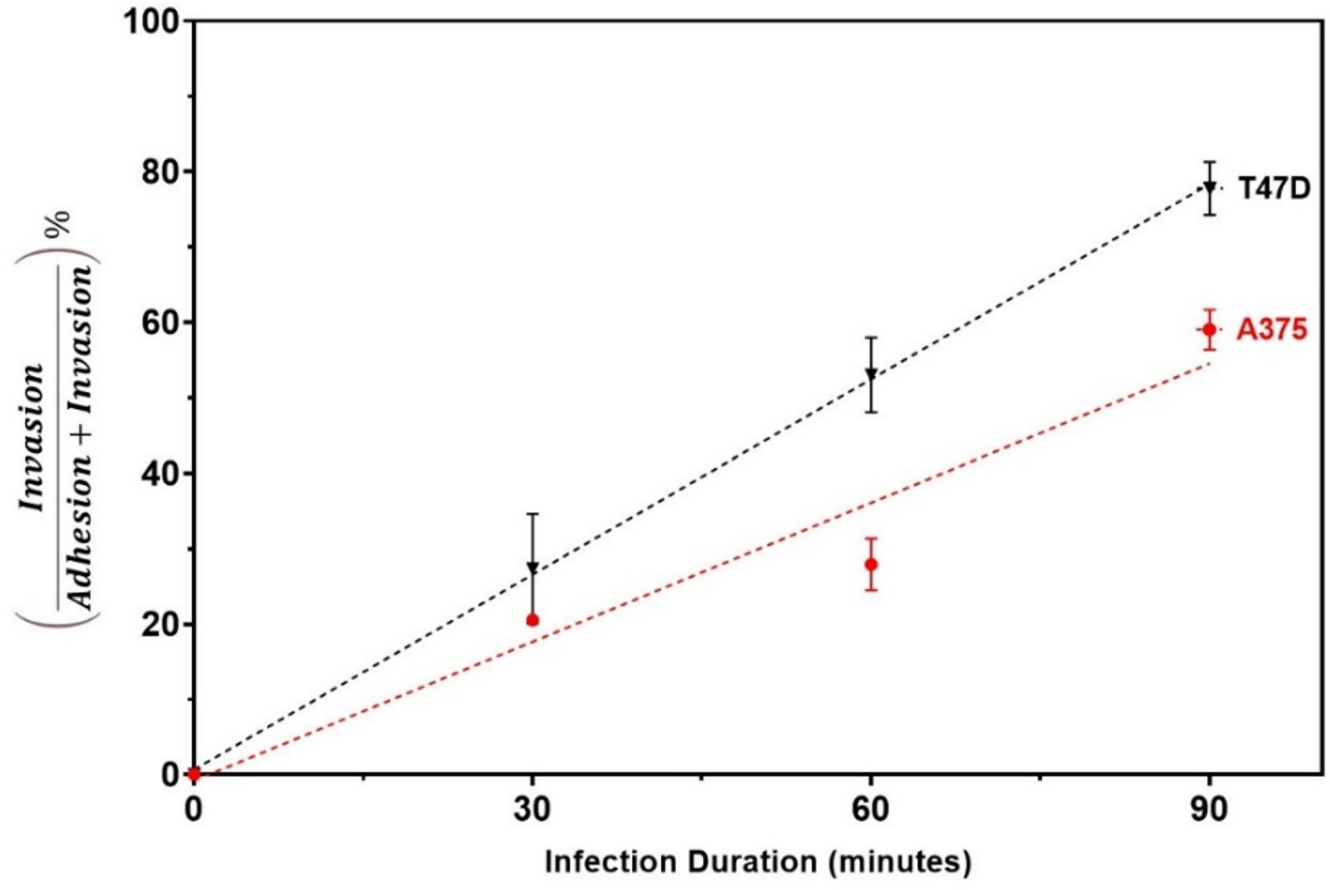
Relative Invasion-to-Adhesion percentage of MRSA bacteria after infecting the two cell lines (melanoma cells; A375, and breast ductal carcinoma cells; T47D) for different durations, 30, 60 and 90 min

In Fig. 5, it can be seen that the number of MRSA bacteria inside T47D breast ductal carcinoma cells, was lower when compared to MRSA bacteria inside A375 melanoma cells. To investigate whether this could be attributed to the difference in MRSA interactions with the two cell lines, or to a variation in the number of initially adherent MRSA bacteria, the relative invasion-to-adhesion percentage (Y-axis) was calculated for each cell line (before laser treatment) at each infection duration (X-axis) is shown in Fig. 6.

As seen in Fig. 6, at each infection duration, MRSA had a higher ability to invade T47D breast ductal carcinoma cells, compared to A375 melanoma cells, even though MRSA initially had a higher ability to adhere to A375 melanoma cells. This observation implies that, while adhesion is an important first step, once MRSA adheres to the cells, the subsequent steps involved in invasion and intracellular survival might be conserved across different cell types, resulting in comparable or even higher invasion ability.

Another observation highlighting the dynamic nature of bacterial infection can be drawn by comparing the number of adherent control samples of MRSA bacteria as the infection duration is prolonged from 30 min. to 60 and 90 min. (highlighted by a descending red arrow across the panels connecting baseline control (0 J/cm²) to anchor comparisons) in Fig. 4, with the number of invaded MRSA bacteria at the same time points (highlighted by an ascending red arrow across the panels connecting baseline control (0 J/cm²) to anchor comparisons)) in Fig. 5. The number of adherent MRSA bacteria decreased while the number of invasive MRSA bacteria increased. This also explains why in Fig. 6, the relative invasion-to-adhesion percentage increased with increasing infection duration (in both human epithelial cell lines). This change could be explained because the bacteria initially adhere to the cell surface and proceed to invade the host cells as time progresses. This temporal shift, where adhesion is an initial step that often leads to invasion, highlights the adaptability of MRSA bacteria in establishing their infections. Once inside the cells, *S. aureus* can exist in a semi-dormant form termed “small colony variants”, making them inherently resistant to antibiotic treatment [6, 54].

Modeling infection to study bacterial pathogenesis in in vitro cellular systems is a straightforward technique to draw accurate and clear conclusions. It depends on delivering a specific number of bacterial pathogens, termed the MOI, to the cultured cells, preferably the host cell type affected during infection [12]. Bacterial pathogens commonly target epithelial or endothelial cells due to their roles as biological barriers and their widespread presence in the body [55–57]. In the current study, we used two human epithelial cancer cell lines, melanoma (A375) and breast ductal carcinoma (T47D), to evaluate MRSA adhesion and invasion ability due to the unavailability of normal epithelial cell lines in our laboratory. Despite the differences between normal and cancer cell lines, many previous studies have also employed cancer cell lines of various origins to study bacterial adhesion and invasion [58–64]. The MOI is critical; if too many bacteria are used the host cells may die quickly, while if too few an infection may not occur [65]. In our experiment, the number of viable bacteria was reduced following laser exposure, so we adjusted the MOI of the control to 200 CFU/cell. This adjustment was a tradeoff, as it increased the likelihood of autoagglutination within the control sample, which hindered the adhesion process [66, 67]. Still, this MOI allowed us to effectively compare the different laser treatments. High-dose vancomycin (200 µg/mL) was used to eliminate extracellular MRSA during the 1-hour incubation. While vancomycin primarily targets growing bacteria, concentrations ≥ 200 µg/mL exhibit rapid bactericidal effects even against non-replicating cells [68], ensuring complete extracellular killing in our assays.

### Comparative evaluation of femtosecond laser-based aPDT for mitigating MRSA adhesion to and invasion of human epithelial cells

To confirm that any variation in the viability of MRSA after each femtosecond laser treatment is not the cause influencing the attenuation of MRSA adhesion and invasion abilities, a comparative evaluation of the adhesion and invasion percentages calculated relative to the initial viability for both control and treated groups following each femtosecond laser treatment was conducted. While log reduction (CFU/mL) is the standard for bactericidal effects, we reported adhesion/invasion suppression as percentages to highlight parameter-dependent virulence attenuation. For example, two laser settings could show similar log reductions (e.g., both 0.5-log) but vastly different adhesion/invasion suppression (e.g., 50% vs. 90%). Percentages capture this nuance better.

**Fig. 7:**
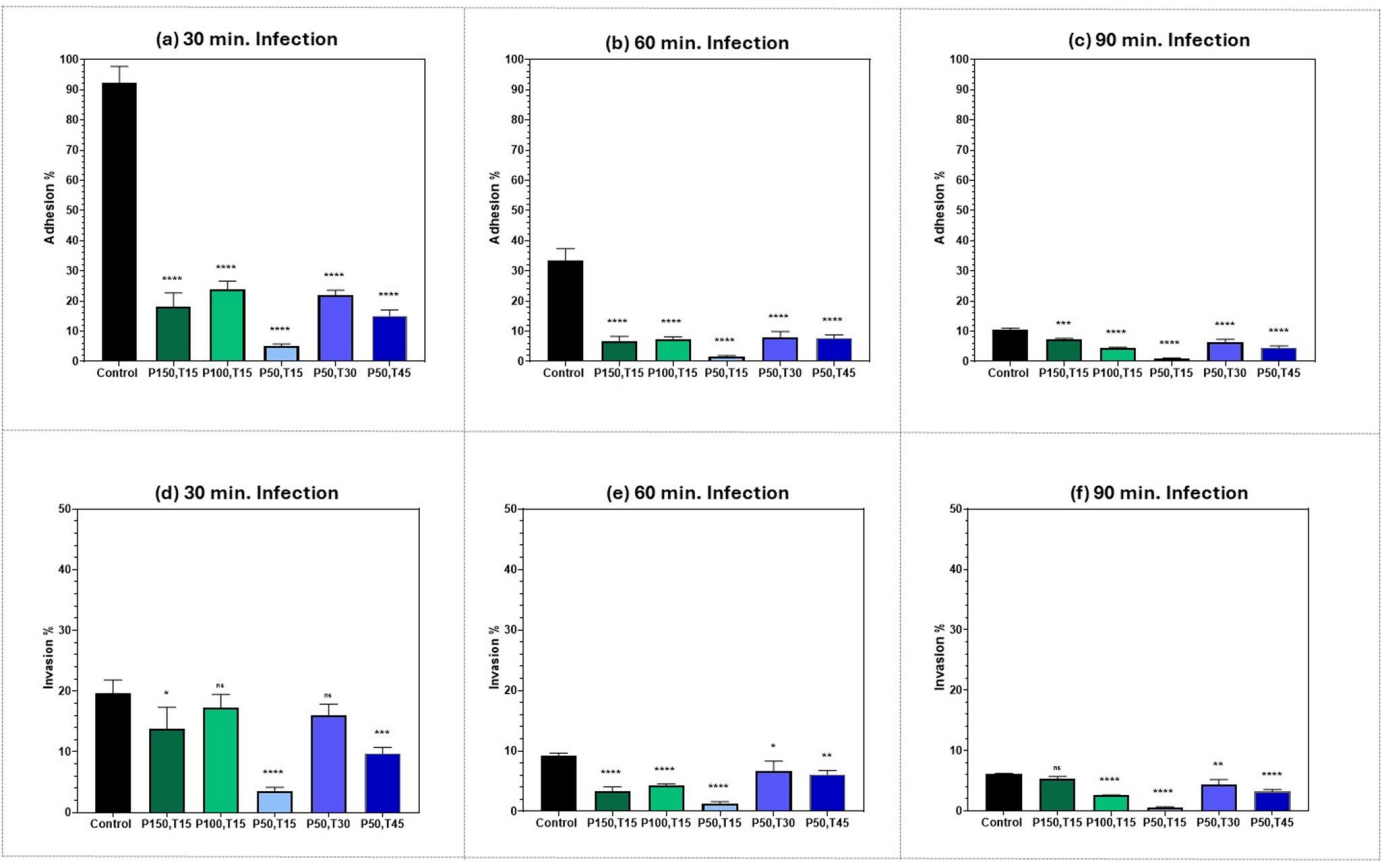
Bar graph of the percentage of adherent, (**a**), (**b**), & (**c**), as well as invasive, (**d**), (**e**), & (**f**), MRSA bacteria in A375 melanoma cells, at three different infection durations: 30, 60, and 90 min., after exposure to 400 nm femtosecond laser irradiation using six sets of parameters: control: no laser; P150,T15: average power of 150 mW for 15 min., P100,T15: average power of 100 mW for 15 min., P50,T15: average power of 50 mW for 15 min., P50,T30: average power of 50 mW for 30 min., P50,T45: average power of 50 mW for 45 min. Statistical significance was determined using ANOVA followed by Tukey’s test (**** *p* < 0.0001, *** *p* < 0.001 ** *p* < 0.01, * *p* < 0.05)

**Fig. 8:**
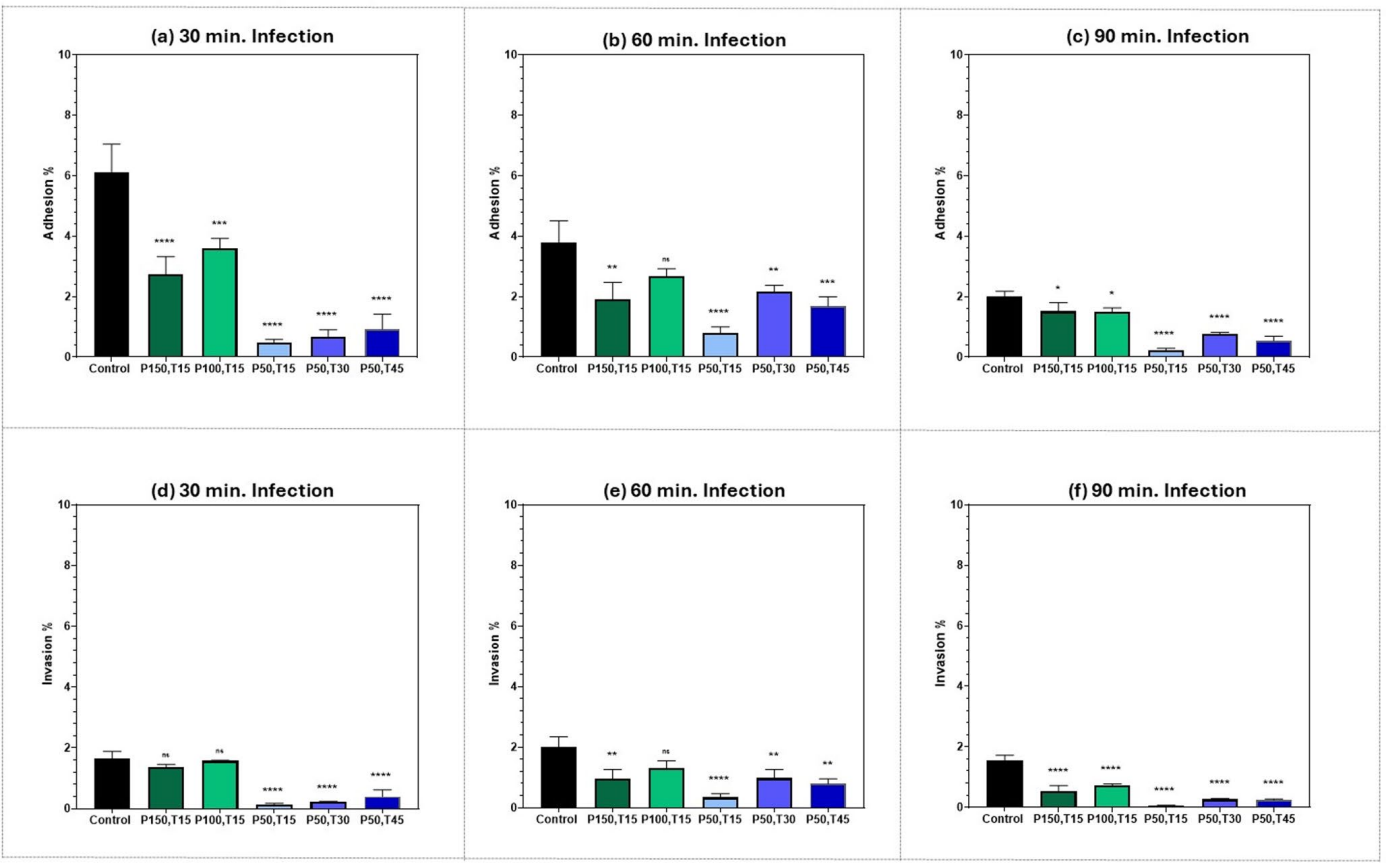
Bar graph of the percentage of adherent, (**a**), (**b**), & (**c**), as well as invasive, (**d**), (**e**), & (**f**), MRSA bacteria in T47D breast ductal carcinoma cells, at three different infection durations: 30, 60, and 90 min., after exposure to 400 nm femtosecond laser irradiation using six sets of parameters: control: no laser; P150,T15: average power of 150 mW for 15 min., P100,T15: average power of 100 mW for 15 min., P50,T15: average power of 50 mW for 15 min., P50,T30: average power of 50 mW for 30 min., P50,T45: average power of 50 mW for 45 min. Statistical significance was determined using ANOVA followed by Tukey’s test (**** *p* < 0.0001, *** *p* < 0.001 ** *p* < 0.01, * *p* < 0.05)

In Fig. 7, we compared the percentage of adherent, (a-c) and invasive (d-f), MRSA bacteria (Y-axis) that infected A375 melanoma cells, after exposure to each femtosecond laser regimen (X-axis), after three different infection durations: 30, 60, and 90 min. The same comparison was made in Fig. 8, for T47D. breast ductal carcinoma cells. As shown in both Figs. 7 and 8, there was a significant reduction in MRSA adhesion and invasion abilities for all femtosecond laser treatments compared to control samples.

**Fig. 9:**
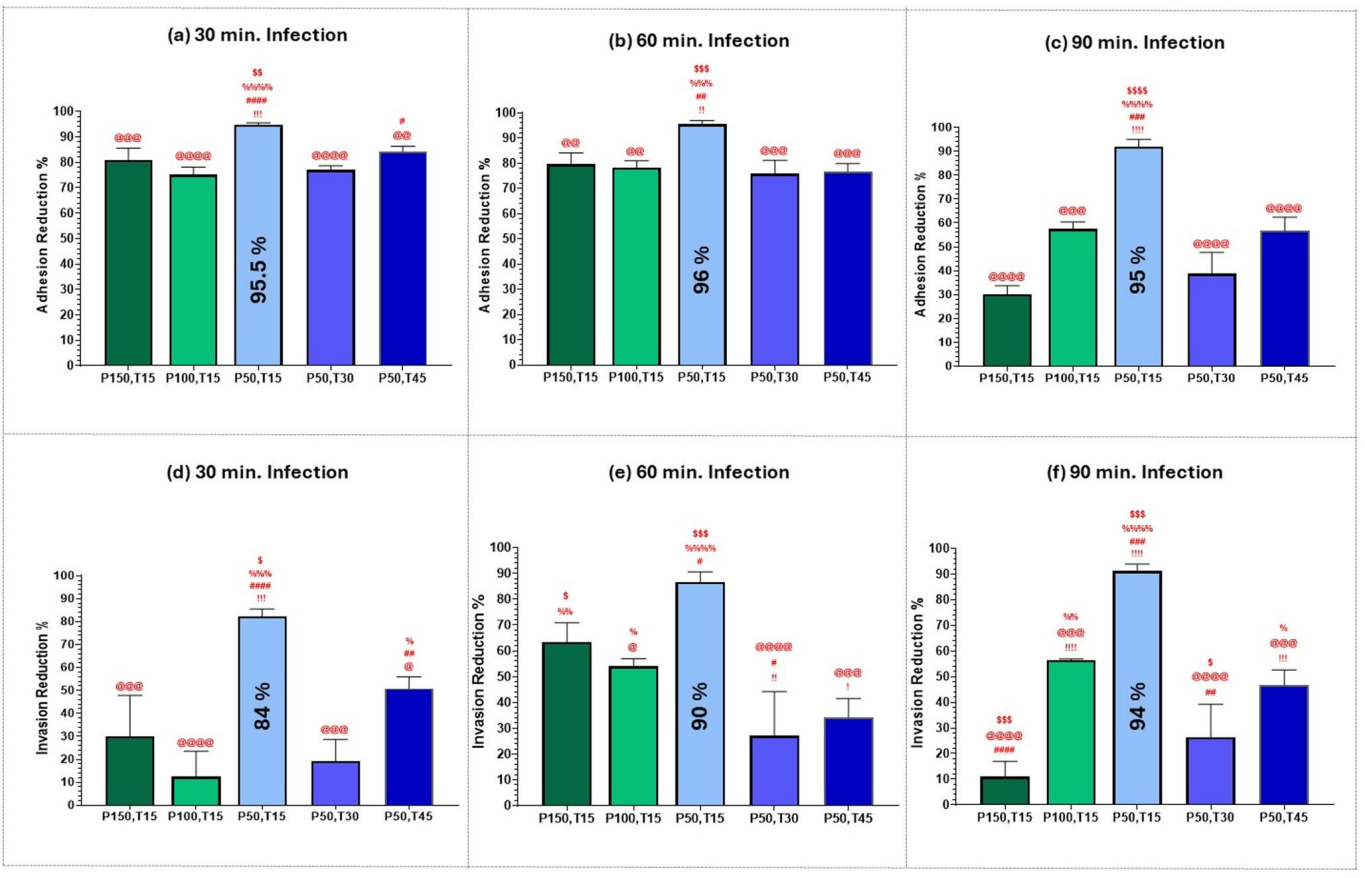
Bar graph of the percentage reduction of adherent, (**a**), (**b**), & (**c**), and invasive, (**d**), (**e**), & (**f**), MRSA bacteria infecting A375 melanoma cells, at three different infection durations: 30, 60, and 90 min., after exposure to 400 nm femtosecond laser irradiation with various parameters: P150,T15: average power of 150 mW for 15 min., P100,T15: average power of 100 mW for 15 min., P50,T15: average power of 50 mW for 15 min., P50,T30: average power of 50 mW for 30 min., P50,T45: average power of 50 mW for 45 min. Statistical significance was determined using ANOVA followed by Tukey’s test. Symbols denote Tukey’s test results:! (vs. P150, T15), # (vs. P100, T15), @ (vs. P50, T15), % (vs. P50, T30) and $ (vs. P50, T45). (!!!!, ####, @@@@, %%%%, ) *p* < 0.0001, (!!!, ###, @@@, %%%, $$$) *p* < 0.001, (!!, ##, @@, %%, $$) *p* < 0.01, (!, #, @, %, $) *p* < 0.05)

**Fig. 10:**
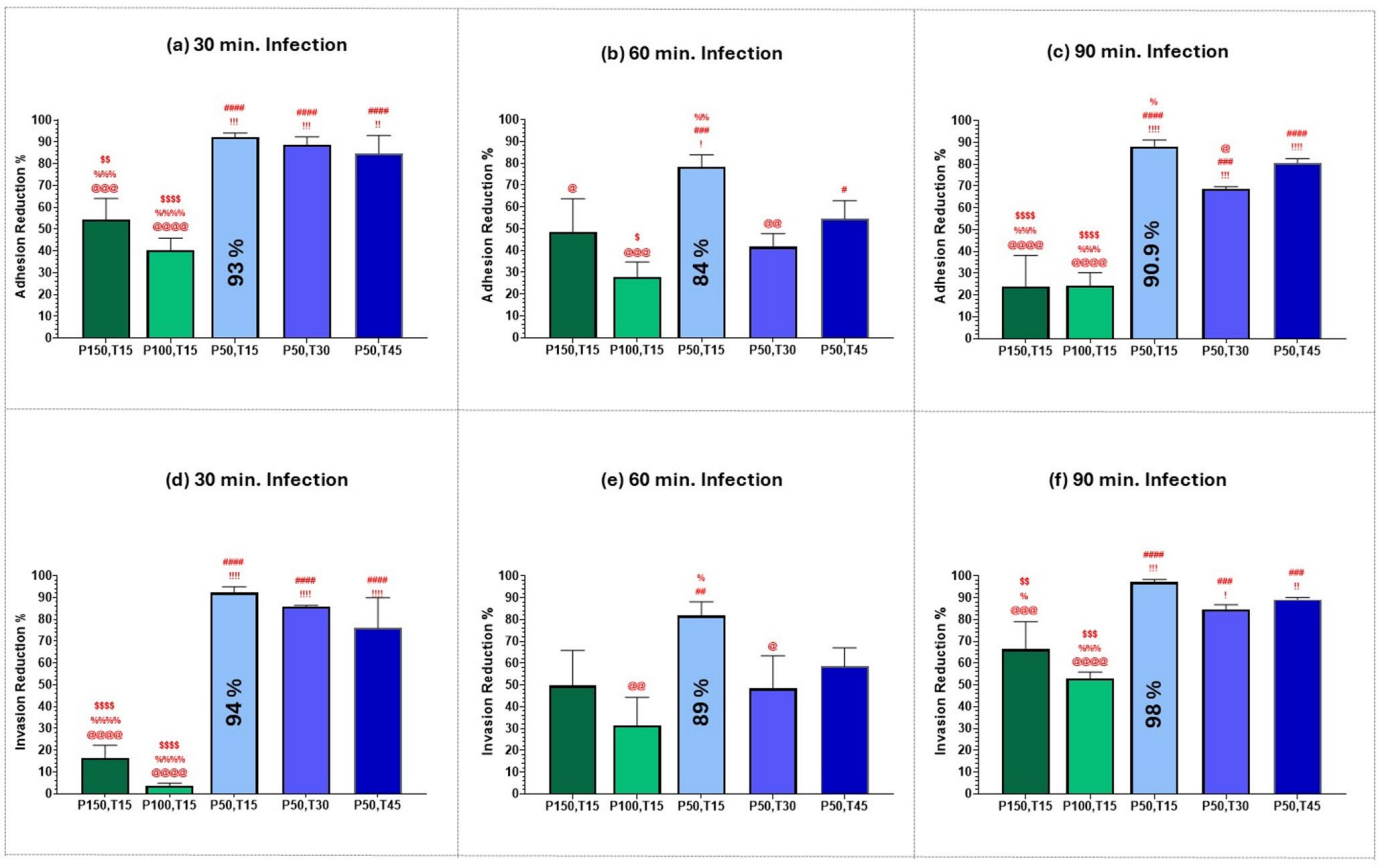
Bar graph of the percentage reduction of adherent, (**a**), (**b**), & (**c**), and invasive, (**d**), (**e**), & (**f**), MRSA bacteria infecting T47D breast ductal carcinoma cells at three different infection durations: 30, 60, and 90 min., after exposure to 400 nm femtosecond laser irradiation with various parameters: P150,T15: average power of 150 mW for 15 min., P100,T15: average power of 100 mW for 15 min., P50,T15: average power of 50 mW for 15 min., P50,T30: average power of 50 mW for 30 min., P50,T45: average power of 50 mW for 45 min. Statistical significance was determined using ANOVA followed by Tukey’s test. Symbols denote Tukey’s test results:! (vs. P150,T15), # (vs. P100,T15), @ (vs. P50,T15), % (vs. P50,T30) and $ (vs. P50,T45). (!!!!, ####, @@@@, %%%%, ) *p* < 0.0001, (!!!, ###, @@@, %%%, $$$) *p* < 0.001, (!!, ##, @@, %%, $$) *p* < 0.01, (!, #, @, %, $) *p* < 0.05)

Next, we calculated the optimum femtosecond laser treatment for the maximum attenuation of MRSA adhesion and invasion at each infection duration (30, 60, and 90 min.). The comparison shown in Fig. 9 involves A375 melanoma cells, and in Fig. 10 involves T47D breast ductal carcinoma cells.

As seen in Figs. 9 and 10, the greatest reduction in both adhesion and invasion of MRSA bacteria was obtained with an average power of 50 mW for 15 min., even though exposure durations of 30 and 45 min. resulted in lower initial viability as seen in Fig. 3. This observation further supports the hypothesis that the efficacy of sublethal femtosecond laser-based aPDT treatment in attenuating MRSA pathogenesis is mainly because of femtosecond laser treatment itself rather than a reduction in bacterial viability. By optimizing laser exposure parameters, we could reduce MRSA virulence without necessarily killing the bacteria themselves.

Targeting bacterial virulence without directly killing the bacterial pathogens could have many advantages. (1) killing bacterial pathogens triggers an abrupt immune response that can lead to inflammation and tissue damage; on the other hand, disarming bacteria of their virulence factors may result in a milder immune response, reducing host cell damage. Especially in severe infections or in immunocompromised individuals. (2) by not exerting life-or-death pressure, anti-virulence strategies are less likely to induce antibiotic resistance in bacterial pathogens [69, 70]. (3) anti-virulence strategies can effectively target both Gram-positive and Gram-negative pathogens with minimal effects on normal host bacterial flora [71].

*S. aureus* expresses numerous surface proteins that facilitate its adhesion to and invasion of host cells. Each of these proteins are differentially expressed with a unique shape and function. These proteins may also bind to specific tissues at different infection stages, making identifying the most critical ones challenging. Thus, despite the considerable advances in understanding how individual bacteria interact with and influence host responses, many questions remain unanswered, making bacterial adhesion and invasion a continuing challenge in biomedical research [72]. It is not a practical strategy to construct a specific vaccine for each of these different adhesins and invasins, which underscores the need for broad-spectrum approaches such as laser-based aPDT, offering promising therapeutic efficacy across a spectrum of pathogens [12]. Exposure to sublethal doses of laser-based aPDT results in stressful growth conditions that might alter bacterial adhesion and invasion processes by inducing stress responses that modify the expression or function of these adhesion and invasion-related proteins. The therapeutic success of aPDT is influenced by the irradiation parameters of the light source, such as mode of action (continuous wave or pulsed), intensity, fluence, exposure duration, and, most critically, wavelength. These parameters substantially affect the photon-tissue interactions, the target photosensitizer that receives the photon energy, the dose of this energy, and the rate of energy transfer [73]. The advancements in pulsed laser system technology, particularly femtosecond lasers, which can deposit energy very quickly into a microscopic volume without affecting the surrounding tissue, are expected to revolutionize laser-based treatment approaches [74]. Femtosecond laser-based aPDT has many advantages, such as minimal absorption by biological samples, precise confinement, low phototoxicity, and clean, noninvasive, controllable laser pulses of non-ionizing radiation [75, 76]. While femtosecond lasers can induce nonlinear optical effects at high peak intensities, our experimental conditions (0.63 GW/cm² peak power density, 100 fs pulses, 6 mm spot size) remained below the threshold for significant multiphoton processes in biological samples [77–79].

The intracellular production of ROS after exposure to sublethal doses of laser-based aPDT can affect bacterial adhesion and invasion in several ways: (1) weakening the necessary structural integrity required for adhesion and invasion by directly damaging bacterial cell membranes; (2) compromising the integrity and functionality of the surface adhesins, invasins, and other CWA proteins; (3) altering the expression of these proteins or disrupting the bacterial signaling pathways that regulate their expression.

However, targeting bacterial virulence factors requires a comprehensive understanding of their mechanisms. Bacteria carefully regulate the expression of these factors through cell-to-cell chemical signaling between bacterial cells and between bacteria and the host cells, using sophisticated regulatory networks known as quorum-sensing systems [25]. The possible alteration of these networks after femtosecond laser exposure will be the focus of our future investigations.

## Conclusion and future perspectives

This study has provided a new perspective on the effect of femtosecond laser-based aPDT in mitigating MRSA infections by targeting virulence factors without compromising bacterial viability. When bacterial pathogens are exposed to sublethal doses of aPDT, the resulting increased oxidative stress inside the bacterial cells may not be sufficient to cause bacterial death, but microbial virulence factors and their expression could still be affected We comparatively evaluated the femtosecond laser-based aPDT exposure parameters for optimum attenuation of bacterial adhesion and invasion abilities. We found that exposure to a femtosecond laser at 400 nm with an average power of 50 mW for 15 min. effectively impaired MRSA adhesion and invasion without much bacterial killing. With the evident biological advantages of using a femtosecond laser as a light source in aPDT, this could be a future clinical approach against multidrug-resistant bacterial infections.

## Data Availability

No datasets were generated or analysed during the current study.
